# Role of follicle-stimulating hormone receptor polymorphism in predicting ovarian response to letrozole in women with polycystic ovary syndrome during ovulation induction

**DOI:** 10.3389/fendo.2025.1585655

**Published:** 2025-06-19

**Authors:** Xiuxian Zhu, Qiaoling Wang, Jingwen Lang, Yunqing Zhi, Yonglun Fu, Shihua Bao

**Affiliations:** ^1^ Shanghai Key Laboratory of Maternal Fetal Medicine, Shanghai Institute of Maternal-Fetal Medicine and Gynecologic Oncology, Shanghai First Maternity and Infant Hospital, School of Medicine, Tongji University, Shanghai, China; ^2^ Department of Reproductive Immunology, Shanghai First Maternity and Infant Hospital, School of Medicine, Tongji University, Shanghai, China; ^3^ Department of Assisted Reproductive Medicine, Shanghai First Maternity and Infant Hospital, School of Medicine, Tongji University, Shanghai, China

**Keywords:** letrozole, ovulation induction, polycystic ovary syndrome, follicle-stimulating hormone polymorphism, ovarian response

## Abstract

**Background:**

Letrozole is a first-line treatment for anovulatory infertility in women with polycystic ovary syndrome (PCOS). Despite its widespread use, a significant proportion of patients exhibit non-responsive cycles, limiting treatment efficacy. Identifying predictive markers for ovarian response could enhance the efficiency and cost-effectiveness of ovulation induction. Previous studies have suggested an association between follicle-stimulating hormone receptor (FSHR) polymorphisms and ovarian response, though the precise relationship between these polymorphisms and ovarian response to letrozole remains poorly defined.

**Methods:**

This retrospective study analyzed data from 133 women with PCOS (93 responsive to letrozole and 40 resistant) between January 2021 and May 2024. Demographic data were collected, and genotyping of FSHR single nucleotide polymorphisms (c.2039A>G, rs6166 at position 680, and c.919A>G, rs6165 at position 307) was performed using predesigned TaqMan SNP assays. A binary logistic regression model was employed to evaluate the predictive value of FSHR polymorphisms in letrozole resistance.

**Results:**

The Asn/Asn polymorphism at position 680 was significantly more prevalent in the letrozole-resistant group (57.5%) compared to the letrozole-responsive group (34.41%) [OR: 1.543 (95% CI, 1.046-2.278), P = 0.013]. Similarly, the Thr/Thr polymorphism at position 307 was more common in the resistance group (57.5% vs. 30.11%) [OR: 1.645 (95% CI, 1.120-2.415), P = 0.003]. Participants with either the Asn/Asn or Thr/Thr polymorphism were more likely to exhibit resistance to letrozole. Logistic regression analysis indicated that the Asn/Asn polymorphism was a potential risk factor for letrozole resistance [OR: 5.227 (95% CI, 0.994-27.490), P = 0.051], while the Thr/Thr polymorphism significantly influenced letrozole response [OR: 7.04 (95% CI, 1.394-35.559), P = 0.018].

**Conclusions:**

The Asn/Asn polymorphism at position 680 and the Thr/Thr polymorphism at position 307 of the FSHR gene may serve as potential predictors of ovulatory failure during letrozole therapy in PCOS patients.

## Introduction

Polycystic ovarian syndrome (PCOS) is a prevalent reproductive endocrine disorder affecting women of childbearing age (1), contributing to approximately 80% of anovulatory infertility cases (2). Ovulation induction is the primary treatment for women with PCOS seeking to conceive (3, 4). However, ovarian responses to induction vary considerably among individuals, complicating the ability to predict successful ovulation. Identifying reliable predictive markers for ovarian response could improve treatment efficiency, reduce costs, and shorten the time to conception.

Follicle-stimulating hormone (FSH) is essential for follicular maturation and the recruitment of the dominant follicle (5). Its action is mediated by the FSH receptor (FSHR), a G protein-coupled receptor expressed exclusively on granulosa cells in the ovary. The FSHR gene, located on chromosome 2p21-p16, consists of 9 introns and 10 exons (6). Several polymorphisms have been identified in the FSHR gene, with two major variants under extensive study. One is a single nucleotide polymorphism (SNP) at position 680 within the intracellular domain, where asparagine (Asn) or serine (Ser) can occupy the position (c.2039A>G, rs6166). The second SNP occurs at position 307 in the extracellular domain, where threonine (Thr) or alanine (Ala) may be present (c.919A>G, rs6165) (7). Studies have shown that the FSHR genotype significantly influences ovarian response during stimulation (8), with the presence of the Ser680 allele in the FSHR gene correlating with ovulation failure following clomiphene citrate (CC) treatment in PCOS patients undergoing ovulation induction (9).

“CC resistance” refers to the failure of ovulation despite CC treatment. For these patients, letrozole is often used, achieving ovulation rates ranging from 54.6% to 84.4% (10, 11). Letrozole functions by inhibiting the conversion of androgens to estrogens, alleviating the negative estrogen feedback on the hypothalamus (12). This results in increased pituitary FSH secretion, thereby promoting follicular development. As a third-generation aromatase inhibitor with a shorter half-life, letrozole has fewer anti-estrogenic effects and side effects compared to CC, making it a preferred option in clinical practice due to its higher ovulation and pregnancy rates (13, 14). Despite its effectiveness, a portion of cycles remain non-responsive to letrozole during ovulation induction (13–15), a phenomenon known as “letrozole resistance” (16–19). To date, the relationship between FSHR polymorphisms and ovarian response to letrozole remains underexplored. This study aims to investigate whether FSHR polymorphisms could serve as a predictive factor for ovarian response to letrozole in women with PCOS undergoing ovulation induction therapy.

## Materials and methods

### Study design and participants

Women diagnosed with PCOS and undergoing ovulation induction therapy with letrozole at the Department of Assisted Reproductive Medicine, Shanghai First Maternity and Infant Hospital, between January 2021 and May 2024, were included in this study. PCOS diagnosis followed the Rotterdam criteria (20), requiring at least two of the following three features: oligomenorrhea or anovulation, clinical or biochemical hyperandrogenism, and the sonographic appearance of polycystic ovaries. Oligomenorrhea was defined as menstrual cycles occurring every 35 days to 3 months, while amenorrhea was defined as cycles exceeding 3 months. Hyperandrogenemia was diagnosed either clinically (e.g., acne or hirsutism) or biochemically (testosterone ≥ 2.5 nmol/L or free androgen index [FAI] ≥ 5). Polycystic ovaries were characterized by the presence of more than 12 antral follicles (2–9 mm in diameter) on ultrasound, or the presence of at least one ovary with a volume greater than 10 cm^3^, without accompanying cysts. Exclusion criteria were (1): age ≥ 40 years, (2) use of other pharmacologic agents for ovulation induction, such as metformin, CC, or gonadotropins, during the study. The study received approval from the Research Ethics Committee of Shanghai First Maternity and Infant Hospital.

### Interventions

In this study, all patients underwent ovulation induction with letrozole alone. Letrozole (Letrozole Tablets; Yimeishu, Zhejiang Hi Sun Pharmaceutical Co., Ltd., Taizhou, China) was administered at a dose of 5 mg per day from day 2 to day 4 of the menstrual cycle (MC) or following progestin-induced withdrawal bleeding for a total of 5 consecutive days. Follicular development was monitored *via* transvaginal ultrasound every 2–4 days after the last dose of letrozole to assess the number of growing follicles and endometrial thickness. Serum levels of FSH, luteinizing hormone (LH), estradiol (E_2_), and progesterone were measured using a chemiluminescent assay (Abbott Biologicals B.V., Weesp, The Netherlands). Ovulation was confirmed by the disappearance of a follicle greater than 14 mm in diameter or a serum progesterone level exceeding 3 ng/mL, followed by either a positive pregnancy test or menstruation (18, 19).

Letrozole resistance was defined as the absence of a dominant follicle (diameter > 10 mm), E_2_ < 70 pg/mL, and progesterone < 1.0 ng/mL 14 days after completing treatment. In these cases, dydrogesterone (Duphaston; Abbott Biologicals B.V., Weesp, The Netherlands) was administered to induce withdrawal bleeding. Blood samples were collected after the 5-day letrozole treatment and stored at -80°C for subsequent analysis.

### Detection of FSHR genotypes

Genomic DNA was extracted from blood samples using the Blood DNA Extraction Kit (EMERTHER Biotech, Shanghai, China). Genotyping of two FSHR SNPs was performed using pre-designed TaqMan SNP assays: c.919A>G (rs6165) with primers (forward: TGCTATACTGGATCTGAGATGTTGA; reverse: CTTAGGGGAGCAGGTCACG) and c.2039A>G (rs6166) with primers (forward: TGCCAACCCCTTCCTCTATG; reverse: GCTCTTTGTGACATACCCTTCAA). Sequencing was conducted using a 3730xl sequencer, and the results were analyzed with DNA Sequencing Analysis software, interpreted using Sequencing Analysis 5.2.0, and aligned using Sequencher 5.1 software.

### Statistical analysis

Data are presented as median (IQR) or n (%) values. The Mann-Whitney U test was used for continuous variables due to non-normal distribution. Fisher’s exact test or Pearson’s chi-square test was employed for categorical variables. Spearman’s correlation analysis assessed the association between letrozole resistance and other variables. Binary logistic regression models were used to evaluate the impact of FSHR polymorphisms on letrozole resistance: model 1 (letrozole resistance as the dependent variable and FSHR variant as the independent variable) and model 2 (letrozole resistance as the dependent variable, with FSHR variant, BMI, and menstrual cycle as independent variables). All statistical analyses were performed using SPSS version 26 (IBM Corporation, Armonk, NY, USA). A two-tailed P value of less than 0.05 was considered statistically significant.

## Results

### Baseline characteristics of participants

A total of 289 women with PCOS who received letrozole treatment at the Department of Assisted Reproductive Medicine, Shanghai First Maternity and Infant Hospital, between January 2021 and May 2024, were initially screened for inclusion. Following screening, 156 women were excluded based on the following criteria: 6 women aged over 40 years, 11 women receiving concomitant pharmacologic agents for ovulation induction, and 139 women who did not provide blood samples for genetic analysis.

**Figure 1:**
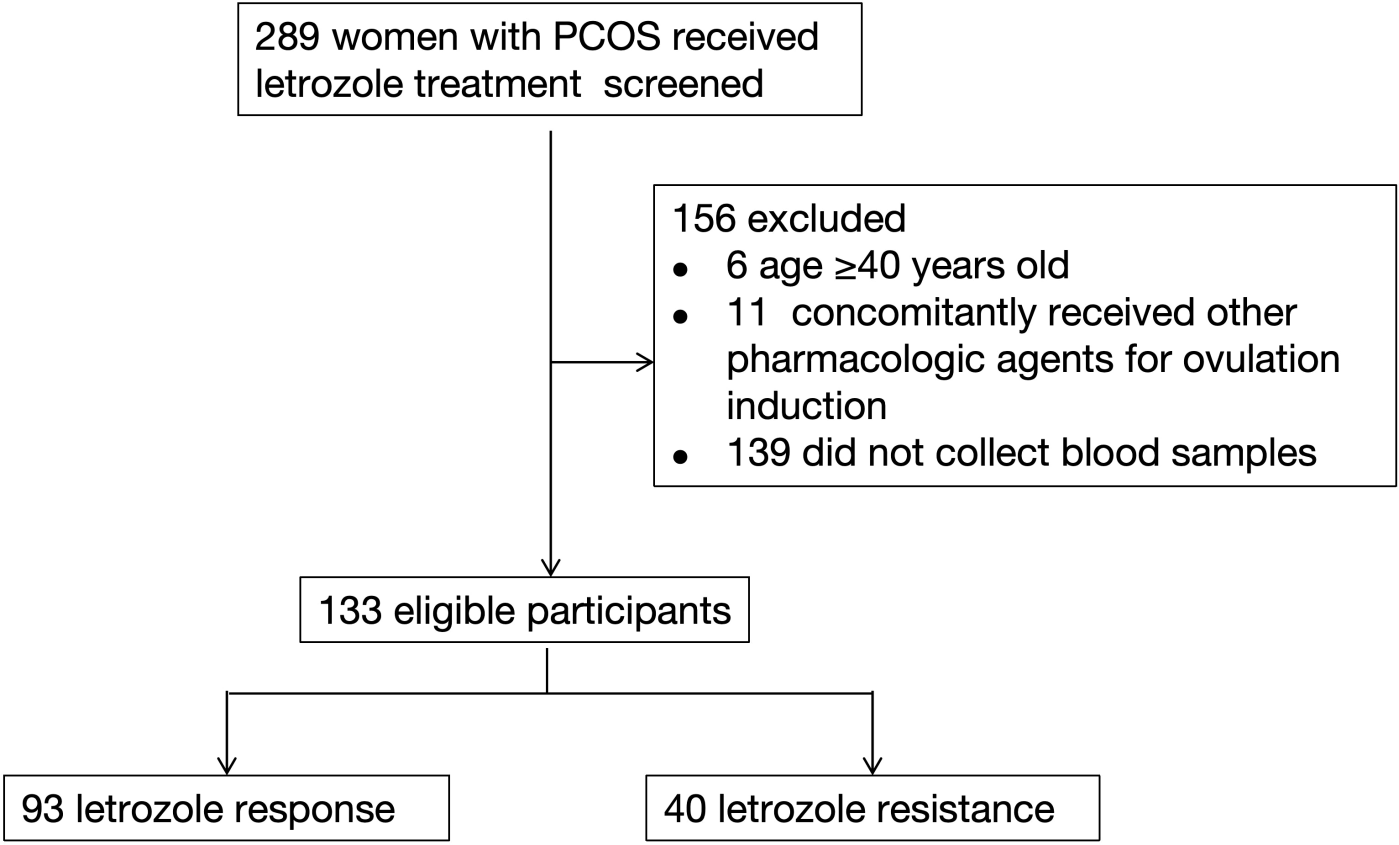
Flowchart illustrating the participant screening process.

Thus, 133 women with PCOS meeting the inclusion criteria were included in the final study cohort. Of these, 93 were classified into the letrozole response group, and 40 into the letrozole resistance group (**Figure 1**). No significant differences were observed between the two groups in terms of age, infertility duration, infertility type, or number of antral follicles (**Table 1**). Basal levels of FSH, LH, E_2_, progesterone, testosterone, and anti-Müllerian hormone (AMH) were also similar across the groups. However, BMI was significantly higher in the letrozole resistance group [26.71 (22.82-28.68)] compared to the response group [23.44 (20.90-26.71)] (P = 0.002), and the menstrual cycle differed significantly between the groups (P = 0.007). All patients exhibited abnormal menstrual cycles and polycystic ovaries on ultrasound, with only one woman diagnosed with biochemical hyperandrogenism.

**Table 1:** Basal characteristics of PCOS patients with letrozole response and resistance.

Characteristic	Letrozole response (n = 93)	Letrozole resistance (n = 40)	P value
Age (year)^*^	29.00 (27.50-32.00)	30.50 (27.25-32.75)	0.304
BMI (kg/m^2^)^*^	23.44 (20.90-26.71)	26.71 (22.82-28.68)	**0.002**
Menstrual cycle, n (%)^†^			**0.007**
Oligomenorrhea	67 (72.04%)	19 (47.5%)	
(35 days-3 months)			
Amenorrhea	26 (27.96%)	21 (52.5%)	
(> 3 months)			
Infertility duration (years)^*^	2 (1-3)	2 (1-3)	0.144
Type of infertility, n (%)^†^			0.288
Primary infertility	71 (76.34%)	27 (67.5%)	
Secondary infertility	22 (23.66%)	13 (32.5%)	
Basal endocrine^*^			
FSH (IU/L)	6.02 (5.25-7.45)	5.84 (5.03-7.44)	0.626
LH (IU/L)	9.04 (5.37-13.23)	7.52 (5.84-9.69)	0.217
E2 (pg/ml)	43.99 (32.92-59.78)	41.48 (36.76-52.08)	0.886
P (ng/ml)	0.55 (0.45-0.70)	0.56 (0.44-0.89)	0.461
T (ng/ml)	0.35 (0.27-0.47)	0.37 (0.29-0.44)	0.721
AMH (ng/ml)	8.90 (6.30-11.98)	9.23 (6.63-12.79)	0.802
Number of antral follicles^*^	30 (27-40)	35 (29.5-40)	0.257
Polycystic ovaries on ultrasound (n)	93	40	
Clinical hyperandrogenism (n)	0	0	
Biochemical hyperandrogenism (n)	1	0	

Data are presented as median (IQR) or n (%). ^*^The Mann-Whitney U test was used for continuous variables due to the non-normal distribution of the data. ^†^Pearson’s Chi-Square test was applied to categorical variables. Abbreviation: BMI, body mass index; FSH, follicle-stimulating hormone; LH, luteinizing hormone; E_2_, estradiol; T, testosterone; P, progesterone; AMH, anti-Müllerian hormone.

Boldface values denote statistical significance (p < 0.05).

### Genotype distributions of FSHR polymorphisms

**Figure 2:**
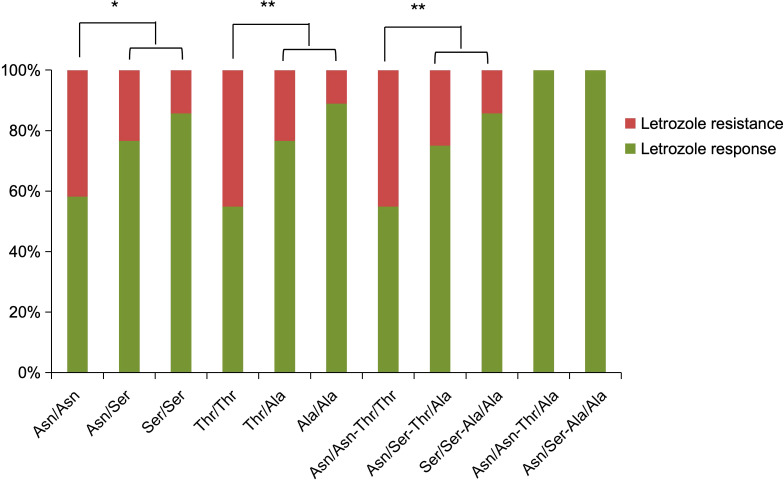
Distribution of FSHR polymorphisms in the letrozole response and resistance groups, expressed as percentages.

FSHR polymorphisms at rs6166 (Asn680Ser) and rs6165 (Thr307Ala) were analyzed. The baseline characteristics of participants with different FSHR genotypes did not differ significantly (**Table 2**). For rs6166 (Asn680Ser), the frequency of the Asn/Asn polymorphism was significantly higher in the letrozole resistance group (57.5%) compared to the response group (34.41%) [OR: 1.543 (95% CI, 1.046-2.278), P = 0.013] (**Table 3**). No significant difference was observed in the distribution of Asn/Ser and Ser/Ser polymorphisms between the groups. For rs6165 (Thr307Ala), the Thr/Thr polymorphism was more frequent in the letrozole resistance group (57.5%) compared to the response group (30.11%) [OR: 1.645 (95% CI, 1.120-2.415), P = 0.003]. No significant differences were found for the other two polymorphisms. Thus, participants with the Asn/Asn or Thr/Thr polymorphisms were more likely to exhibit resistance to letrozole than those with the Asn/Ser or Ser/Ser polymorphisms or the Thr/Ala or Ala/Ala polymorphisms (**Figure 2**).

**Table 2:** Characteristics of PCOS patients with different FSHR genotypes.

Characteristic	rs6166(Asn680Ser)A/G				rs6165(Thr307Ala)A/G			P value
	Asn/Asn (n = 55)	Asn/Ser (n = 64)	Ser/Ser (n = 14)	P value	Thr/Thr (n = 51)	Thr/Ala (n = 64)	Ala/Ala (n = 18)	
Age (year) ^*^	30 (29-33)	29 (27-31.75)	28.5 (27-34.25)	0.167	30 (29-33)	29 (27-32)	28 (27-31)	0.105
BMI (kg/m^2^) ^*^	24.30 (22.40-27.55)	23.62 (20.93-27.30)	24.30 (20.83-26.71)	0.748	24.20 (22.40-27.55)	24.13 (21.37-27.30)	23.67 (20.30-26.71)	0.666
Menstrual cycle, n (%)^†^				0.471				0.939
Oligomenorrhea	37 (67.3%)	42 (65.6%)	7 (50%)		33 (64.7%)	42 (65.6%)	11 (61.1%)	
(35 days-3 months)								
Amenorrhea	18 (32.7%)	22 (34.4%)	7 (50%)		18 (35.3%)	22 (34.4%)	7 (38.9%)	
(> 3 months)								
Infertility duration (years) ^*^	2 (1-3)	2 (1-3)	1.5 (1-2.25)	0.189	1 (1-3)	2 (1-3)	2 (1-3)	0.399
Type of infertility, n (%)^†^				0.279				0.560
Primary infertility	43 (78.2%)	47 (73.4%)	8 (57.1%)		40 (78.4%)	46 (71.9%)	12 (66.7%)	
Secondary infertility	12 (21.8%)	17 (26.6%)	6 (42.9%)		11 (21.6%)	18 (28.1%)	6 (33.3%)	
Basal endocrine								
FSH (IU/L) ^*^	5.99 (5.01-7.25)	5.96 (5.30-7.50)	6.52 (5.63-7.73)	0.391	5.99 (4.74-7.25)	5.96 (5.16-7.56)	6.52 (5.65-7.45)	0.407
LH (IU/L) ^*^	7.22 (5.50-10.26)	8.68 (4.67-12.38)	13.13 (5.59-15.74)	0.134	7.22 (5.41-10.26)	8.68 (5.72-12.38)	12.08 (4.86-15.58)	0.177
E_2_ (pg/ml) ^*^	45.53 (34.13-54.26)	43.54 (32.74-60.71)	36.44 (29.88-54.98)	0.506	46.10 (34.13-54.26)	42.92 (32.74-60.55)	38.42 (30.18-56.92)	0.773
P (pg/ml) ^*^	0.57 (0.45-0.76)	0.56 (0.44-0.74)	0.49 (0.45-0.62)	0.431	0.56 (0.45-0.77)	0.56 (0.45-0.73)	0.49 (0.43-0.62)	0.473
T (ng/ml) ^*^	0.34 (0.27-0.44)	0.35 (0.28-0.46)	0.40 (0.21-0.51)	0.813	0.35 (0.27-0.44)	0.36 (0.28-0.47)	0.35 (0.21-0.47)	0.621
AMH (ng/ml) ^*^	9.26 (7.25-14.03)	9.32 (5.46-11.84)	7.91 (4.73-11.17)	0.188	9.53 (7.25-13.89)	8.90 (5.70-11.98)	8.20 (5.26-11.19)	0.404
Number of sinusoidal follicles^*^	32 (28-40)	30 (26.75-40)	32 (29-43.5)	0.451	32 (28-40)	30 (27-40)	32 (26.5-43.5)	0.585
Polycystic ovaries on ultrasound (n)	55	64	14		51	64	18	
Clinical hyperandrogenism (n)	0	0	0		0	0	0	
Biochemical hyperandrogenism (n)	0	1	0		0	1	0	

Data are presented as median (IQR) or n (%). ^*^The Kruskal-Wallis test was used for continuous variables due to the non-normal distribution of the data. ^†^Pearson’s Chi-Square test was applied to categorical variables. Abbreviation: BMI, body mass index; FSH, follicle-stimulating hormone; LH, luteinizing hormone; E_2_, estradiol; T, testosterone; P, progesterone; AMH, anti-Müllerian hormone.

**Table 3:** Genotype distributions of FSHR polymorphisms in PCOS patients with letrozole response and resistance.

Genotype	Letrozole response (n = 93)	Letrozole resistance (n = 40)	OR (95% CI)	P value
rs6166(Asn680Ser)A/G				
Asn/Asn (%)	34.41% (32)	57.5% (23)	1.543 (1.046-2.278)	**0.013**
Asn/Ser (%)	52.69% (49)	37.5% (15)	0.757 (0.549-1.044)	0.108
Ser/Ser (%)	12.90% (12)	5% (2)	0.917 (0.825-1.019)	0.145
rs6165(Thr307Ala)A/G				
Thr/Thr (%)	30.11% (28)	57.5% (23)	1.645 (1.120-2.415)	**0.003**
Thr/Ala (%)	52.69% (49)	37.5% (15)	0.757 (0.549-1.044)	0.108
Ala/Ala (%)	17.20% (16)	5% (2)	0.872 (0.775-0.979)	0.059
Genotype combination				
Asn/Asn-Thr/Thr (%)	30.11% (28)	57.5% (23)	1.645 (1.120-2.415)	**0.003**
Asn/Ser-Thr/Ala (%)	48.39% (45)	37.5% (15)	0.826 (0.605-1.126)	0.247
Ser/Ser-Ala/Ala (%)	12.90% (12)	5% (2)	0.917 (0.825-1.019)	0.173
Asn/Asn-Thr/Ala (%)	4.30% (4)	0		0.234
Asn/Ser-Ala/Ala (%)	4.30% (4)	0		0.234

Data are presented as n (%) or 95% CI. Fisher’s exact test or Pearson’s Chi-Square test was used for categorical variables.

Boldface values denote statistical significance (p < 0.05).

### Prediction of FSHR polymorphisms for letrozole resistance

**Figure 3:**
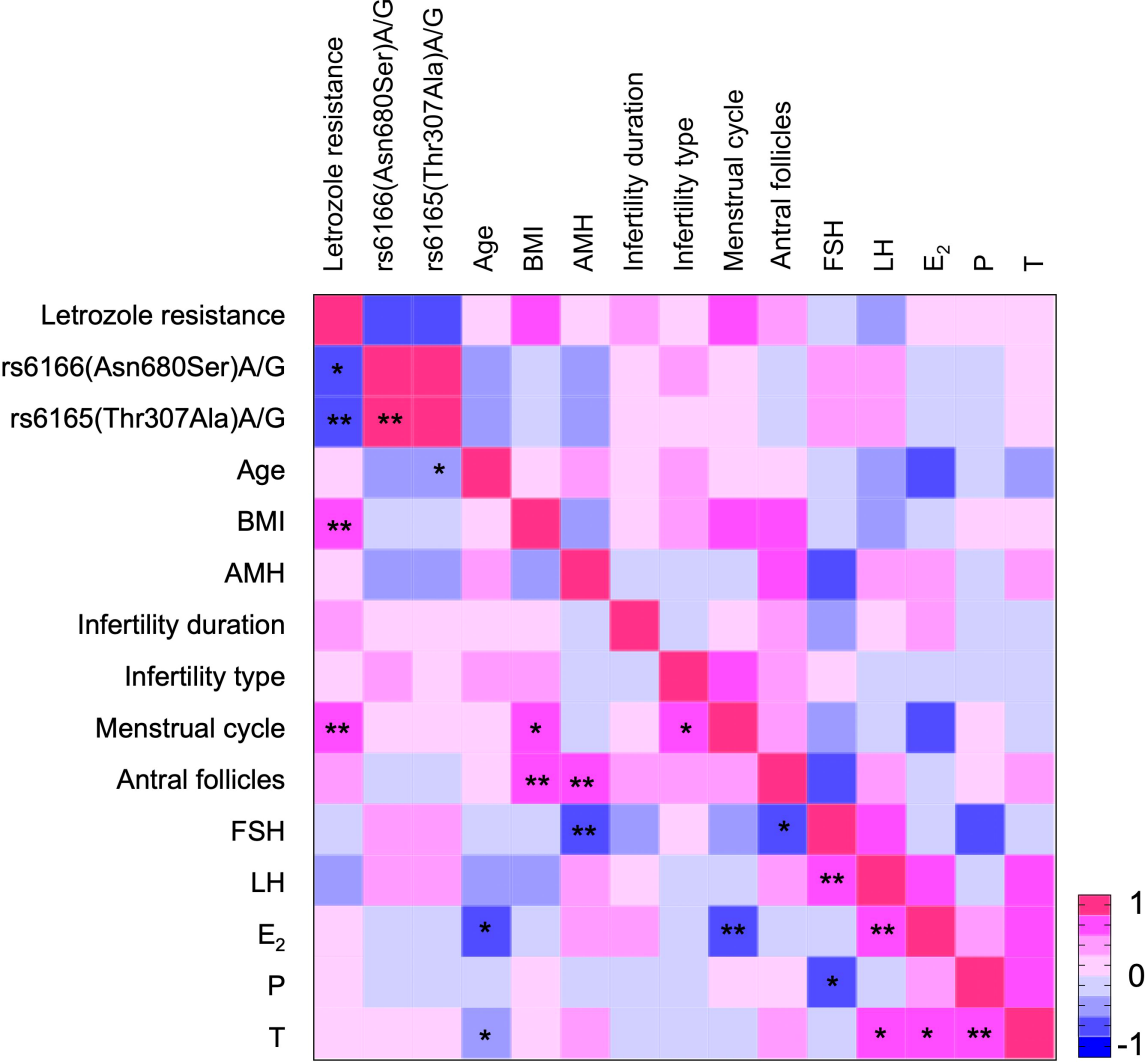
Heatmap showing the association between letrozole resistance and various variables. Red indicates a positive correlation, while blue denotes a negative correlation. The intensity of the color reflects the magnitude of the correlation coefficient. *P < 0.05, **P < 0.01, indicating statistical significance of the correlation coefficient.

Spearman correlation analysis indicated that letrozole resistance was significantly associated with FSHR polymorphisms and positively correlated with both BMI and menstrual cycle (**Figure 3**). Logistic regression analysis was conducted with letrozole resistance as the dependent variable and FSHR variant as a predictive factor (Model 1, **Table 4**). The analysis showed that the Asn/Asn polymorphism tended to affect letrozole response [OR: 4.312 (95% CI, 0.879-21.146), P = 0.072], while the Thr/Thr polymorphism significantly influenced the response [OR: 6.571 (95% CI, 1.367-31.588), P = 0.019]. Given that BMI and menstrual cycle may serve as potential confounders for letrozole resistance, a further analysis using Model 2 was conducted, incorporating these factors as independent variables. The results were consistent with those obtained in Model 1, reinforcing the finding that the Thr/Thr polymorphism is a sensitive predictor for letrozole resistance.

**Table 4:** Prediction of FSHR polymorphisms on letrozole resistance.

Model		OR	95% CI	P value
Model 1				
Dependent variable	Letrozole resistance			
Predictive variable	Asn/Asn	4.312	0.879-21.146	0.072
	Asn/Ser	1.837	0.369-9.141	0.458
	Ser/Ser	1		
	Thr/Thr	6.571	1.367-31.588	**0.019**
	Thr/Ala	2.449	0.505-11.886	0.266
	Ala/Ala	1		
Model 2				
Dependent variable	Letrozole resistance			
Predictive variable	Asn/Asn	5.227	0.994-27.490	0.051
	Asn/Ser	2.005	0.380-10.581	0.412
	Ser/Ser	1		
	Thr/Thr	7.04	1.394-35.559	**0.018**
	Thr/Ala	2.387	0.471-12.097	0.294
	Ala/Ala	1		
Independent variable	BMI			
	Menstrual cycle			

Data are presented as 95% CI and analyzed using a binary logistic regression model.

Boldface values denote statistical significance (p < 0.05).

## Discussion

This study represents the first investigation into whether FSHR polymorphisms can predict ovarian response to letrozole in women with PCOS during ovulation induction. The data reveal that participants with the Asn/Asn polymorphism at position 680 or the Thr/Thr polymorphism at position 307 of FSHR were significantly more likely to experience ovulatory failure following letrozole treatment. Among these, the Thr/Thr polymorphism emerged as the most sensitive predictor of such failure.

FSHR polymorphisms have been extensively studied in both PCOS and non-PCOS populations, with genotype distribution varying across different ethnic groups (21, 22). In this study, the distribution of FSHR genotypes in women with PCOS was 41.35% Asn/Asn, 48.12% Asn/Ser, and 10.53% Ser/Ser at position 680, and 38.35% Thr/Thr, 48.12% Ala/Thr, and 13.53% Ala/Ala at position 307. These results align with those reported in Japanese (23) and South Korean (24) populations but diverge from findings in Caucasian women (22). Kim et al. observed a significant difference in FSHR genotype distribution between women with PCOS and age-matched controls, suggesting an association between FSHR polymorphisms and PCOS susceptibility. Specifically, the genotype distribution in the PCOS group was 39.5%, 47.2%, and 13.3% for Asn/Asn, Asn/Ser, and Ser/Ser, respectively, compared to 46.4%, 45.4%, and 8.2% in controls; and 38.5%, 46.7%, and 14.9% for Thr/Thr, Thr/Ala, and Ala/Ala in the PCOS group, compared to 46.6%, 45.4%, and 8.0% in controls (24). However, this increased susceptibility was not confirmed in Chinese and Thai populations (25). Valkenburg et al. reported that the Ser680 allele correlated with higher levels of FSH, LH, and testosterone, as well as a higher frequency of hyperandrogenism, but was not associated with PCOS risk itself (26). This suggests that while FSHR polymorphisms may affect hormonal levels, their role in PCOS pathogenesis remains uncertain. The impact of FSHR variants on basal FSH levels has been observed in several studies (8, 27). In contrast, our study observed no significant association between FSHR genotypes and basal sex hormone levels, consistent with the findings of Overbeek et al. (9).

The relationship between FSHR polymorphisms and ovarian response to ovulation induction remains unclear. Although numerous studies have examined the impact of FSHR variants on ovarian response during IVF/ICSI cycles, demonstrating that Ser/Ser and Ala/Ala polymorphisms necessitate higher doses of exogenous FSH for ovarian stimulation (28–34), few have focused on their effect during ovulation induction with drugs like letrozole and CC (9, 26, 35). For instance, Overbeek et al. reported that PCOS patients with the Ser/Ser genotype were more likely to exhibit CC resistance (28%) compared to those with Asn/Ser (14%) or Asn/Asn (15%) genotypes, suggesting that FSHR polymorphisms may influence CC resistance (9). Similarly, Valkenburg et al. found that patients with the Ser/Ser genotype at position 680 had an 89% higher likelihood of CC resistance (OR = 1.9, 95% CI, 1.1–3.3) (26). In contrast, Laven et al. observed no significant differences in ovarian response or required FSH doses among anovulatory women with varying FSHR genotypes at positions 307 and 680 (35), implying that the role of FSHR polymorphisms in ovarian responsiveness may not be straightforward.

In the present study, the Asn/Asn 680 and Thr/Thr 307 polymorphisms were found to be more likely associated with resistance to letrozole treatment, contrasting with previous studies suggesting that Ser/Ser 680 and Ala/Ala 307 polymorphisms are linked to a better response (9, 26, 32). Logistic regression analysis, both unadjusted and adjusted for other factors, confirmed this association between FSHR polymorphisms and letrozole resistance. These results highlight that FSHR genotype plays a critical role in determining ovarian sensitivity to ovulation induction medications. Additionally, even individuals with the same genotype may exhibit varying responses to different treatments.

The underlying cause of this variation remains unclear, but it may be attributed to the distinct mechanisms through which letrozole and CC exert their effects. Unlike CC, which primarily influences the hypothalamic-pituitary axis, letrozole increases ovarian sensitivity to FSH by elevating local androgen levels within the ovaries. This mechanism may explain why patients resistant to CC might respond favorably to letrozole (10, 11). These findings support the potential benefit of tailoring ovulation induction treatments based on FSHR genotype. Specifically, patients with the Ser/Ser or Ala/Ala polymorphism may exhibit a better response to letrozole, while those with the Asn/Asn or Thr/Thr polymorphism might derive greater benefit from CC as a first-line treatment. Such personalized approaches could enhance treatment efficiency, minimize cycle cancellations, and ultimately increase the likelihood of success in a shorter time frame.

This study also identified a significant association between higher BMI and longer menstrual cycles with ovulatory failure following letrozole treatment, aligning with previous research. Palomba et al. found that PCOS patients who failed to ovulate after CC treatment had a significantly higher BMI compared to those who ovulated (32.3 vs. 25.9) (36). Similarly, Overbeek et al. observed significant BMI differences between CC non-responders and responders (26.3 vs. 23.6) (9). Furthermore, a prospective study by Küniger et al. revealed a significant correlation between BMI and the required FSH dose for achieving monofollicular development in CC-resistant PCOS patients (37). These findings suggest that BMI may serve as an independent predictor of ovarian sensitivity in PCOS. After adjusting for BMI and menstrual cycle length, the predictive value of FSHR polymorphisms for resistance to letrozole remained statistically significant, further reinforcing their role in predicting ovarian response to ovulation induction.

This study has several strengths. It is the first to examine the predictive effect of FSHR genotypes on ovarian response to letrozole in women with PCOS, utilizing both Spearman correlation analysis and logistic regression models to validate the results. However, several limitations must be acknowledged. One major limitation is the relatively low prevalence of clinical and biochemical hyperandrogenism in our cohort, which may reflect ethnic differences. In a previous randomized controlled trial (RCT) conducted by our group, a similarly low prevalence of hyperandrogenism was observed among PCOS patients, with only 4.05% (3/74) in the study group and 1.35% (1/74) in the control group presenting with clinical hyperandrogenism (19). This suggests that ethnic factors may influence the manifestation of hyperandrogenism in PCOS, potentially limiting the generalizability of our findings to populations with higher prevalence rates of hyperandrogenism. Further studies involving more diverse populations are necessary to validate these conclusions. Another limitation is the retrospective design of our study and the relatively small sample size, highlighting the need for larger, prospective trials to confirm these findings. Additionally, the underlying mechanisms by which FSHR genotypes affect ovarian response to letrozole remain unclear, warranting further exploration.

## Conclusion

In conclusion, our study provides novel evidence that FSHR polymorphisms at rs6165 (position 307) and rs6166 (position 680) are associated with ovarian response in women with PCOS undergoing ovulation induction with letrozole. Specifically, the Asn/Asn 680 and Thr/Thr 307 polymorphisms may serve as valuable predictors of ovulatory failure. These findings contribute to a deeper understanding of the genetic factors influencing ovulation induction outcomes and highlight the potential for personalized treatment strategies. By considering FSHR genotypes, clinicians can better tailor ovulation induction agents and their dosages, enhancing treatment efficacy, minimizing costs, and ultimately improving patient care for women with PCOS.

## Data Availability

The original contributions presented in the study are included in the article/**Supplementary Material**. Further inquiries can be directed to the corresponding authors.
